# NSUN2-mediated mRNA m^5^C Modification Regulates the Progression of Hepatocellular Carcinoma

**DOI:** 10.1016/j.gpb.2022.09.007

**Published:** 2022-09-30

**Authors:** Dan Song, Ke An, Wenlong Zhai, Luyao Feng, Yingjie Xu, Ran Sun, Yueqin Wang, Yun-Gui Yang, Quancheng Kan, Xin Tian

**Affiliations:** 1 Department of Pharmacy, the First Affiliated Hospital of Zhengzhou University, Zhengzhou 450052, China; 2 CAS Key Laboratory of Genomic and Precision Medicine, Beijing Institute of Genomics, Chinese Academy of Sciences and China National Center for Bioinformation, Beijing 100101, China; 3 Henan Key Laboratory of Precision Clinical Pharmacy, Zhengzhou University, Zhengzhou 450052, China; 4 Department of Hepatobiliary and Pancreatic Surgery, the First Affiliated Hospital of Zhengzhou University, Zhengzhou 450052, China; 5 Key Laboratory of Digestive Organ Transplantation of Henan Province, Zhengzhou University, Zhengzhou 450052, China

**Keywords:** 5-methylcytosine, Hepatocellular carcinoma, NSUN2, Ras pathway, Sorafenib

## Abstract

RNA modifications affect many biological processes and physiological diseases. The **5-methylcytosine** (m^5^C) modification regulates the progression of multiple tumors. However, its characteristics and functions in **hepatocellular carcinoma** (HCC) remain largely unknown. Here, we found that HCC tissues had a higher m^5^C methylation level than the adjacent normal tissues. Transcriptome analysis revealed that the hypermethylated genes mainly participated in the phosphokinase signaling pathways, such as the Ras and PI3K-Akt pathways. The m^5^C methyltransferase **NSUN2** was highly expressed in HCC tissues. Interestingly, the expression of many genes was positively correlated with the expression of *NSUN2*, including *GRB2*, *RNF115*, *AATF*, *ADAM15*, *RTN3*, and *HDGF*. Real-time PCR assays further revealed that the expression of the mRNAs of *GRB2*, *RNF115*, and *AATF* decreased significantly with the down-regulation of *NSUN2* expression in HCC cells. Furthermore, NSUN2 could regulate the cellular sensitivity of HCC cells to **sorafenib** via modulating the Ras signaling pathway. Moreover, knocking down *NSUN2* caused cell cycle arrest. Taken together, our study demonstrates the vital role of NSUN2 in the progression of HCC.

## Introduction

Liver cancer accounts for the sixth most common cancer and the third leading cause of cancer mortality worldwide [1]. The main type of liver cancer is hepatocellular carcinoma (HCC), which is a primary malignant tumor originating from the liver epithelial tissue or mesenchymal tissue [2]. Various kinase-related signaling pathways are aberrantly activated in HCC, such as the Ras/Raf/MAPK/Erk pathway (Ras pathway) and the PI3K/Pten/Akt/mTOR pathway (PI3K-Akt pathway) [3], [4]. The Ras pathway regulates the proliferation, apoptosis, and differentiation of HCC cells [5]. This kinase pathway recruits the GRB2/SHC/SOS complex and promotes the phosphorylation of Ras and Raf when the membrane surface receptor of the epidermal growth factor receptor (EGFR) receives the stimulation signal. Then, a high level of phosphorylated Erk (p-Erk), as an activation marker, translocates into the nucleus and combines with other transcription initiation factors to promote oncogene expression [6]. As the first-line molecular-targeted drug for HCC, sorafenib can specifically inhibit Raf phosphorylation in the Ras pathway and plays an important role in inhibiting HCC cell proliferation and angiogenesis [7], [8].

The 5-methylcytosine (m^5^C) modification occurs in many types of RNAs, including mRNAs and non-coding RNAs. NOP2/Sun RNA methyltransferase (NSUN2) mainly catalyzes the formation of m^5^C as a writer protein [9], induces the differentiation of epidermal and neural stem cells [10], [11], and directly affects gene expression in viruses by regulating the splicing of HIV-1 RNA [12]. Modified RNAs are recognized by Y-box binding protein 1 (YBX1) and Aly/REF export factor (ALYREF). YBX1 and ALYREF promote mRNA stability [13] and nuclear translocation [14] as the readers of m^5^C. No m^5^C eraser has been identified yet, although some proteins are involved in m^5^C oxidation. For example, AlkB homolog 1 (ALKBH1) and ten-eleven translocation (TET) family proteins have been identified as dioxygenases that catalyze the conversion of m^5^C to hm^5^C, which regulates RNA degradation and mitochondrial activity [15], [16].

As a critical RNA m^5^C catalytic enzyme, the functions of NSUN2 have been described in multiple types of cancer. NSUN2 affects the mRNA stability of the heparin-binding growth factor (HDGF) by catalyzing m^5^C modification in its 3′-untranslated region (3′-UTR), which promotes the pathogenesis of bladder cancer [12]. Additionally, NSUN2 is overexpressed in breast cancer (BRCA) and hypopharyngeal squamous cell carcinoma (HPSCC) [17], [18]. Pan-cancer analysis showed that NSUN2 is positively correlated with DNA copy number and mRNA expression, which are associated with poor prognosis [18], [19]. NSUN2 can regulate the m^5^C modification of H19 lncRNA and promote the occurrence and development of HCC by recruiting G3BP stress granule assembly factor 1 (G3BP1) [20]. The m^5^C profiles of circular RNA and mRNA were discovered in HCC [21], [22]. However, the biological significance of NSUN2 and the characteristics of the m^5^C modification in HCC have not been fully investigated.

In this study, we analyzed the characteristics of the mRNA m^5^C modification in HCC tissues compared to those in the adjacent tissues at the single-nucleotide resolution. The mechanism by which NSUN2 regulates the expression of multiple target genes was determined at the bioinformatic and experimental levels. We examined the effect of NSUN2 on regulating HCC cell sensitivity to sorafenib by affecting the activity of the Ras pathway. Additionally, the down-regulation of NSUN2 in HCC cells arrested the cell cycle. The mechanisms of m^5^C regulated by NSUN2 were involved in the progression of HCC.

## Results

### mRNAs are frequently **m^5^**C-hypermethylated in HCC tissues

**Figure 1:**
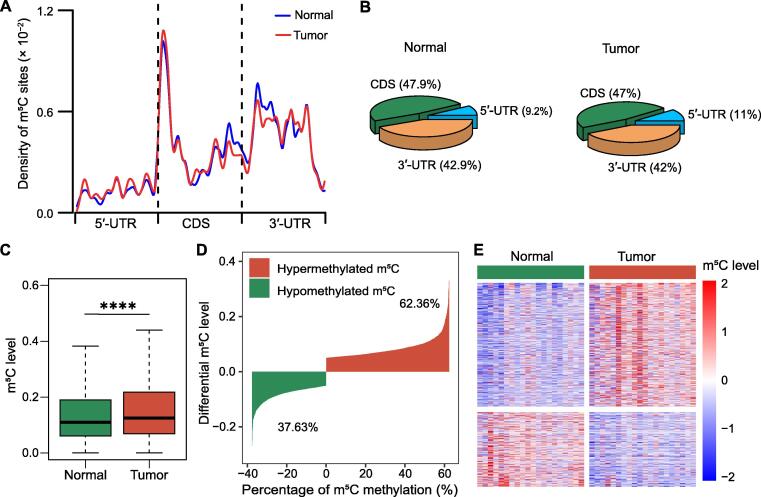
**mRNAs are frequentlym^5^C-hypermethylated in HCC tissuesA.** Distribution pattern of the m^5^C sites on mRNAs in the HCC tissues (tumor) and the adjacent tissues (normal). **B.** Different proportions of m^5^C modifications in regions of mRNA between the HCC tissues and the adjacent tissues. **C.** The overall m^5^C modification level is higher in the HCC tissues than in the adjacent tissues, as determined by the BisSeq data analysis. Statistical significance was calculated by Wilcoxon test (****, *P* = 5.116E−09). **D.** Difference in the m^5^C modification levels between the HCC tissues and the adjacent tissues. **E.** Heatmap showing the differential m^5^C methylation levels between the HCC tissues and the adjacent tissues. HCC, hepatocellular carcinoma; m^5^C, 5-methylcytidine; CDS, coding sequence; UTR, untranslated region.

**Supplementary Figure S1:**
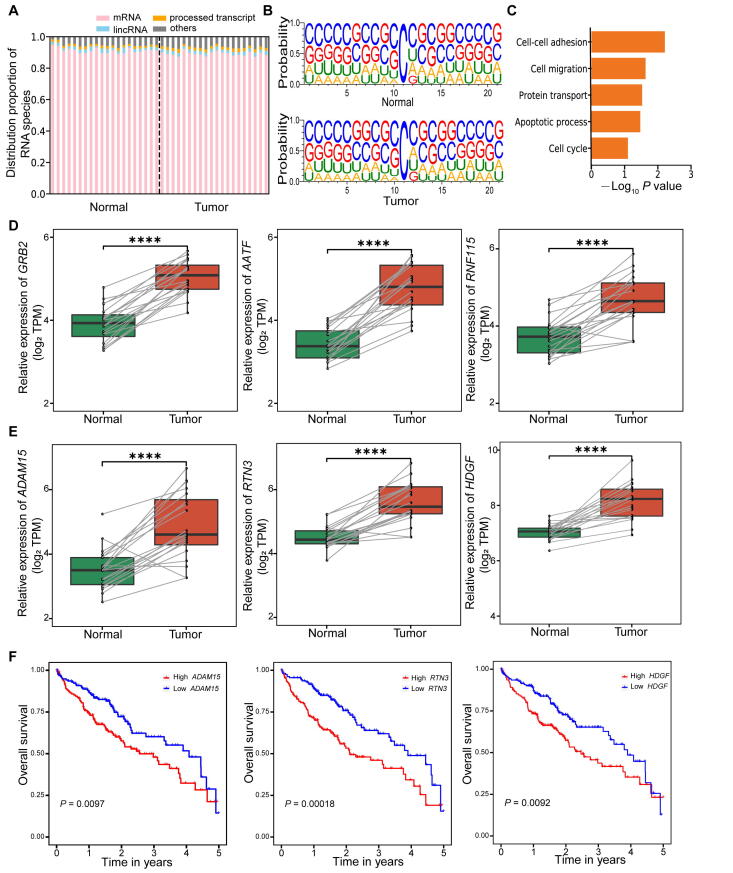
**Distribution characteristics of m5C in HCC and expression pattern of target genes**. **A.** The type and proportion of RNA in the sequencing library. **B.** The sequences are proximal to the mRNA m^5^C sites in HCC tissues and the adjacent tissues. **C.** The m^5^C hypermethylated and highly expressed genes in HCC tissues were involved in different cellular processes, determined by the GO functional analysis. **D.** and **E.** The expression of target genes, including GRB2, AATF, RNF115, ADAM15, RTN3, and HDGF, was higher in HCC tissues than that in the adjacent tissues, determined by transcriptome analysis, ****, *P* < 0.0001. **F.** The overall survival analysis showed that the higher expression of ADAM15, RTN3, and HDGF was correlated with a poor prognosis in HCC patients; ADAM15 (*, *P* = 0.0097), RTN3 (**, *P* = 0.00018), HDGF (*, *P* = 0.0092). TPM, transcripts per kilobase of exon model per million mapped reads; HCC, hepatocellular carcinoma; GO, gene ontology.

To reveal the m^5^C modification features in HCC, we collected 20 pairs of HCC tumor samples and analyzed the transcriptome (RNA sequencing; RNA-seq) data and RNA bisulfite sequencing (RNA-BisSeq) data. The m^5^C sites were enriched in mRNAs in the HCC tumor tissues and the adjacent tissues (Figure S1A). The distribution characteristics of the m^5^C modifications in HCC mRNAs were found to be enriched downstream of the translation initiation site in the mRNA coding sequence (CDS) region (Figure 1A). The distribution pattern was consistent with other mammalian cells previously reported [13]. The proportion of the m^5^C modification in different regions of the mRNA was statistically analyzed. The m^5^C sites covered in the 3′-UTR, CDS, and 5′-UTR were similar in cancer tissues and adjacent tissues, and the CDS region contained the highest number of m^5^C sites (Figure 1B). A sequence frequency logo displayed an embedding feature of m^5^C sites in CG-rich environments (Figure S1B). Our RNA-BisSeq data identified 2482 m^5^C sites in mRNAs with differential methylation levels (as shown in Table S1). Additionally, we found that mRNA m^5^C modification in HCC tissues was significantly higher than that in the adjacent tissues for the overall methylation level (Figure 1C). We found 1548 and 934 sites for hypermethylated and hypomethylated, respectively. The ratio of hypermethylated sites in tumor tissues was 62.36%, and the ratio of hypomethylated sites was 37.63% relative to that in the normal tissues (Figure 1D). The heatmap analysis showed the differential methylation level of the m^5^C sites between the adjacent tissues and HCC tissues (Figure 1E). In summary, mRNAs are frequently m^5^C-hypermethylated in HCC.

### Multiple **m^5^**C-hypermethylated genes related to NSUN2 participate in oncogenic pathways

**Figure 2:**
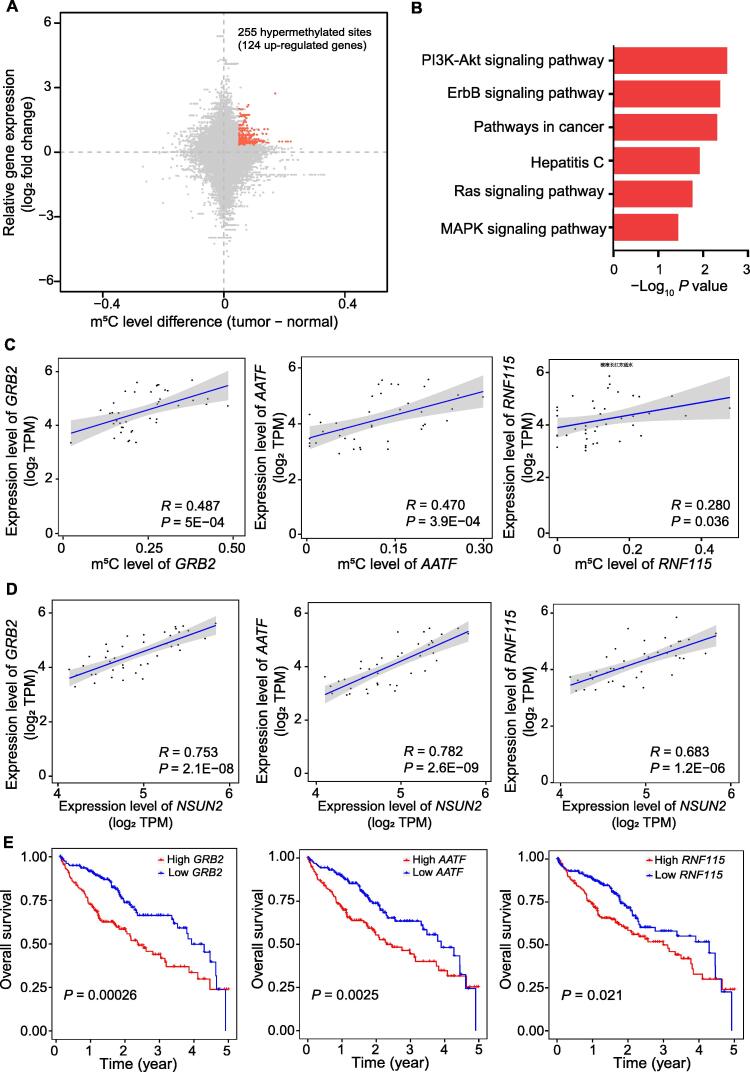
**Multiplem**^**5**^**C-hypermethylated genes related to NSUN2 participate in the oncogenic pathwaysA.** The distribution of mRNAs with a significant change in the m^5^C methylation level and the gene expression level in HCC tissues and the adjacent tissues. **B.** The KEGG analysis showed that m^5^C-hypermethylated genes with high expression levels in the HCC tissues were enriched in oncogenic signaling pathways. **C.** A relation analysis showed that the expression levels of *GRB2*, *RNF115*, and *AATF* were positively correlated with their m^5^C modification levels. **D.** The expression levels of *GRB2*, *RNF115*, and *AATF* were positively correlated with the *NSUN2* expression level. **E.** The overall survival analysis indicates the correlation of the mRNA expression of *GRB2*, *RNF115*, and *AATF* with poor prognosis in HCC patients. *P* values were calculated by Student’s *t*-test. KEGG, Kyoto Encyclopedia of Genes and Genomes; TPM, transcripts per kilobase of exon model per million mapped reads.

To further investigate the effect of m^5^C on the progress of HCC, we identified differentially expressed mRNAs with hypermethylated m^5^C sites in HCC tissues. We detected 255 hypermethylated sites, covering 124 genes with high mRNA expression (Figure 2A). Through the Kyoto Encyclopedia of Genes and Genomes (KEGG) analysis of highly m^5^C-modified genes, several tumor-related pathways, including the PI3K-Akt, ErbB, and Ras signaling pathways, were found to be enriched in m^5^C modifications (Figure 2B). Moreover, these highly m^5^C-modified genes were found to be involved in the progression of cell migration, apoptosis, and cell cycle (Figure S1C). We investigated the genes (*GRB2*, *AATF*, *RNF115*, *ADAM15*, *RTN3*, and *HDGF*) that are highly expressed in HCC and modified by m^5^C (Figure S1D and E). To further determine whether the specific m^5^C-modified genes are regulated by NSUN2 in HCC, first, we analyzed the correlation between the mRNA expression level of target genes and their m^5^C modification level (Figure 2C). Then, we analyzed the correlation between the *NSUN2* mRNA expression and the mRNA expression of the target genes (Figure 2D). The results showed that the mRNA expression of the target genes was related to their m^5^C modification level and also to the *NSUN2* mRNA expression level (Figure 2C and D). Additionally, the results of the TCGA analysis indicated that higher expression levels of these target genes (*GRB2*, *AATF*, *RNF115*, *ADAM15*, *RTN3*, and *HDGF*) were associated with a poor prognosis of HCC (Figure 2E, Figure S1F). The aforementioned results suggest that multiple hypermethylated genes associated with NSUN2 participate in oncogenic pathways.

### NSUN2 is highly expressed in HCC and regulates mRNA **m^5^**C modification

**Figure 3:**
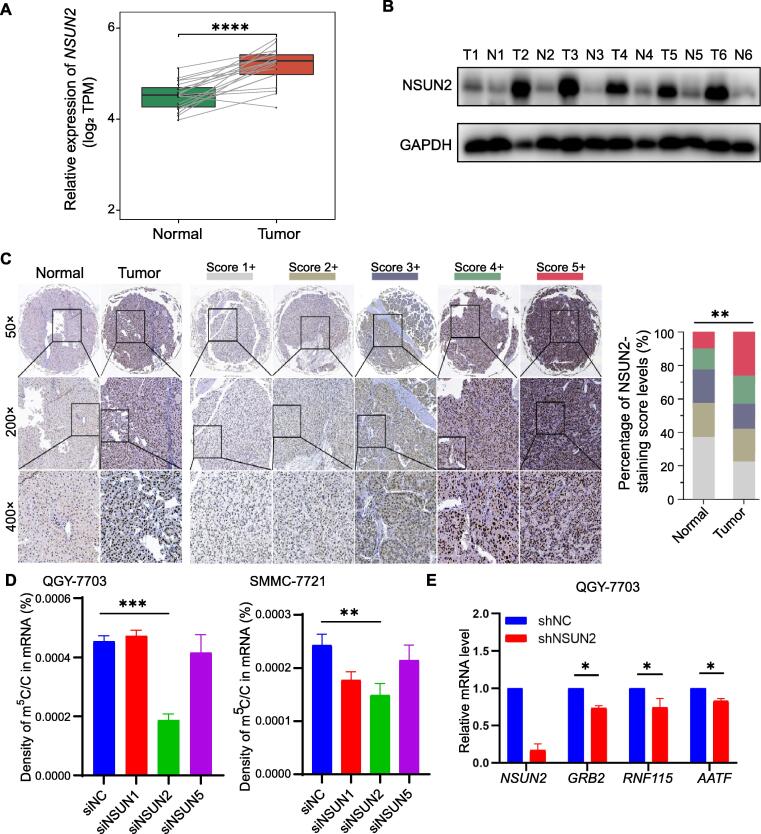
**NSUN2 is highly expressed in HCC and regulates mRNA m^5^C modificationA.** The expression of *NSUN2* mRNA was higher in HCC tissues than in the adjacent tissues determined by transcriptome analysis. **B.** Western blot analysis showed the higher expression of NSUN2 in HCC tissues than in the adjacent tissues. GAPDH was used as a reference control. “T” indicates a tumor smaple, and “N” indicates the adjacent tissue. **C.** Immunohistochemical analysis showed the higher expression of NSUN2 in HCC tissues than in the adjacent tissues. **D.** In HCC cells, UHPLC-MS/MS analysis showed that the down-regulation of *NSUN2* significantly decreased the density of m^5^C/C in mRNAs. **E.** The real-time PCR analysis showed that the mRNA expression of *GRB2*, *RNF115*, and *AATF* was significantly decreased when *NSUN2* was silenced in QGY-7703 cells. Data were represented by mean ± SD. Statistical significance was determined by Student’s *t*-test (*, *P* < 0.05; **, *P* < 0.01; ***, *P* < 0.001; ****, *P* < 0.0001). UHPLC-MS/MS, ultra-high performance liquid chromatography-mass spectrometry/mass spectrometry.

**Supplementary Figure S2:**
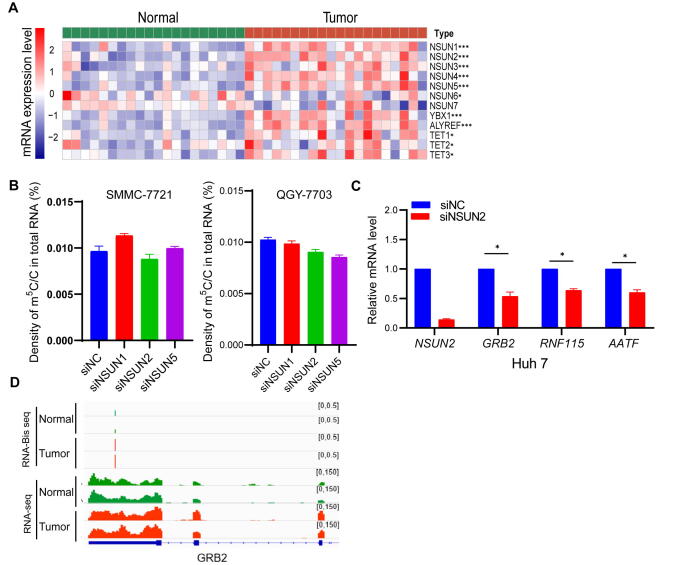
**NSUN2 regulates downstream target gene expression, especially GRB2**. **A.** The mRNA expression of m^5^C writers and readers in HCC tissues was higher than that in the adjacent tissues, determined by the transcriptomic analysis, ***, *P* < 0.001. **B.** In HCC cell lines, the UHPLC-MS/MS analysis showed that downregulation of NSUN2 did not change the density of m5C/C in total RNA. **C.** Real-time PCR analysis showed that the mRNA expression levels of GRB2, RNF115, and AATF were significantly lower in Huh7 *NSUN2* knockdown cells, *, *P* < 0.05; **, *P* < 0.01. **D.** The m^5^C modification in the 3′ UTR and the expression of GRB2 mRNA were analyzed by IGV visualization in HCC tissues and the adjacent tissues. UHPLC-MS/MS, ultra-high performance liquid chromatography-mass spectrometry/mass spectrometry; UTR, untranslated region; IGV, itegrative genomics viewer.

Transcriptome data analysis showed that m^5^C-related writer and reader proteins were highly expressed in HCC tissues (Figure S2A). Previous studies have shown that NSUN2 is involved in the regulation of m^5^C modification and affects tumor progression [20], [23]. Here we focused on the regulatory relationship between NSUN2 and m^5^C-modified target genes in the progression of HCC. Expression data displayed that *NSUN2* mRNA was overexpressed in HCC tissues (Figure 3A). We confirmed the protein expression of NSUN2 in some of the HCC tissue cohorts (*n* = 6) through Western blot (Figure 3B). The results of the immunohistochemical analysis showed that NSUN2 had a higher expression in HCC tissues than that in the adjacent normal tissues (Figure 3C). Additionally, the m^5^C modification level of total RNA and mRNAs in the HCC cell lines (QGY-7703 and SMMC-7721) were analyzed by ultra-high performance liquid chromatography-mass spectrometry/mass spectrometry (UHPLC-MS/MS). We used siRNAs to knock down *NSUN2* and its family members (*NSUN1/5*). NSUN2 was found to be an important methyltransferase for mRNAs in HCC cells (Figure 3D, Figure S2B). Real-time PCR revealed that the mRNA expression levels of *GRB2*, *RNF115*, and *AATF* were significantly decreased in the *NSUN2*-knockdown HCC cells (Figure 3E, Figure S2C). The integrative genomics viewer (IGV) tracks displayed the read coverage of the *GRB2* mRNA in the RNA-seq and RNA-BisSeq data, and showed the up-regulation of m^5^C modifications and mRNA abundance in *GRB2* in HCC tissues compared to that in the adjacent tissues (Figure S2D). We concluded that NSUN2 plays a critical role in regulating the m^5^C modification of the target genes (*GRB2*, *RNF115*, and *AATF*) in HCC.

### NSUN2 affects the sensitivity of HCC cells to sorafenib by regulating the activity of the Ras pathway

**Figure 4:**
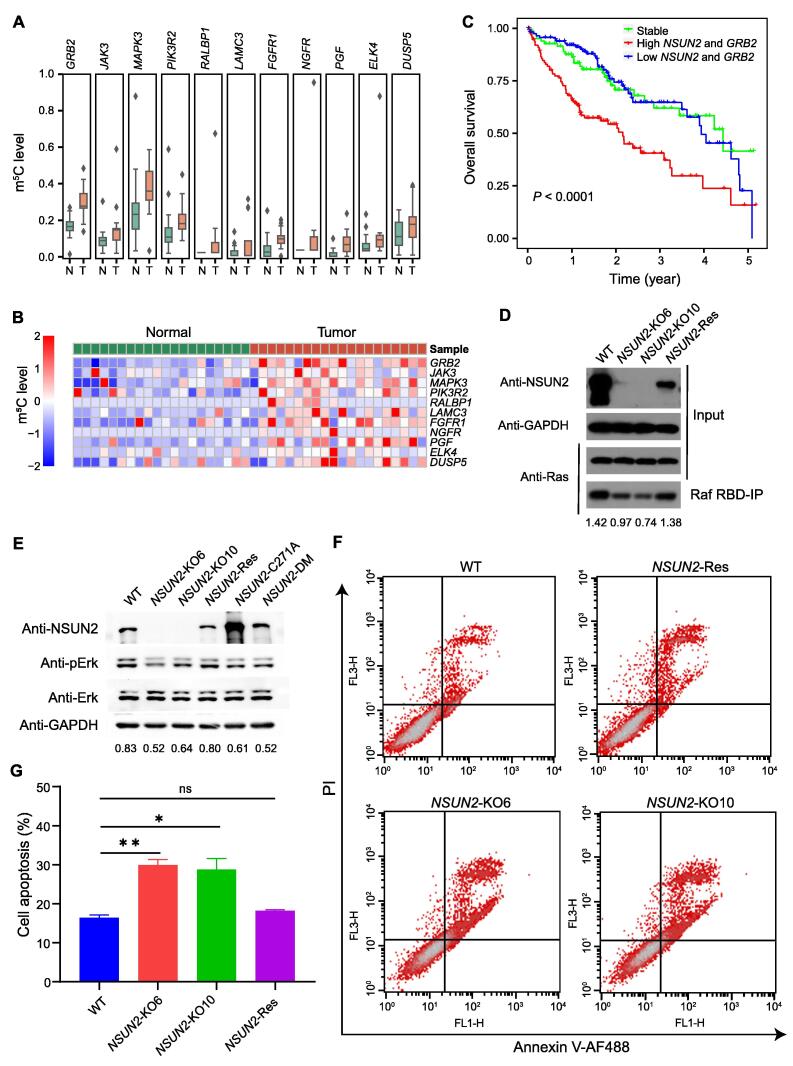
**NSUN2 affects the sensitivity of HCC cells to sorafenib by regulating the activity of the Ras pathwayA.** Box plots showing the mRNA m^5^C levels of the Ras pathway-related genes. **B.** Heatmap showing the differential mRNA m^5^C levels of the Ras pathway-related genes in HCC tissues and the adjacent tissues. **C.** The overall survival analysis indicated that the high expression of *NSUN2* and *GRB2* was correlated with the worst prognosis in HCC patients (****, *P* < 0.0001). **D.** Western blot showing the Ras activity detected in wild-type QGY-7703 cells (WT), *NSUN2*-knockout cells (*NSUN2*-KO6/KO10), and *NSUN2*-rescued cells (*NSUN2*-Res). **E.** Western blot of Erk and p-Erk in *NSUN2*-knockout cells (*NSUN2*-KO6/KO10) and *NSUN2*-rescued cells (*NSUN2*-Res, *NSUN2*-C271A, and *NSUN2*-DM). *NSUN2*-Res, *NSUN2*-C271A, and *NSUN2*-DM indicate wild-type rescued, binding site mutant rescued, and binding site and catalytic site double mutant rescued, respectively. GAPDH was used as a reference control. **F.** Sorafenib treatment and flow cytometry analysis of the apoptosis of QGY-7703 cells when *NSUN2* was knockout or rescued. **G.** The statistical analysis of the apoptosis ratio shown in (F). Data were represented by mean ± SD. Statistical significance was determined by Student’s *t*-test (*, *P* < 0.05; **, *P* < 0.01; ns, not significant). Raf RBD, Ras-binding domain of Raf; IP, immunoprecipitation; p-Erk, phosphorylated-Erk; PI, propidium iodide.

The activity of the Ras pathway is abnormally high in most HCC patients, which leads to a poor prognosis [24]. The phosphorylation of Raf is one of the crucial targets of sorafenib [25]. GRB2 is a critical upstream linker that promotes Raf phosphorylation that is regulated by NSUN2 in esophageal squamous cell carcinoma. A previous study has reported that GRB2 is a key upstream regulator of Raf phosphorylation and is regulated by NSUN2 [26]. To confirm the effect of the regulation of m^5^C by NSUN2 on the activity of the Ras pathway in HCC, the m^5^C modification levels of genes (such as *GRB2*, *MAPK3*, and *PIK3R2*) in the Ras pathway were analyzed. These genes were hypermethylated in HCC tissues (Figure 4A). A heatmap analysis showed that the m^5^C modification levels of these genes were increased in HCC tissues (Figure 4B). According to the results of the TCGA data analysis, HCC patients with higher expression of *NSUN2* and *GRB2* had the worst prognosis (Figure 4C).

**Supplementary Figure S3:**
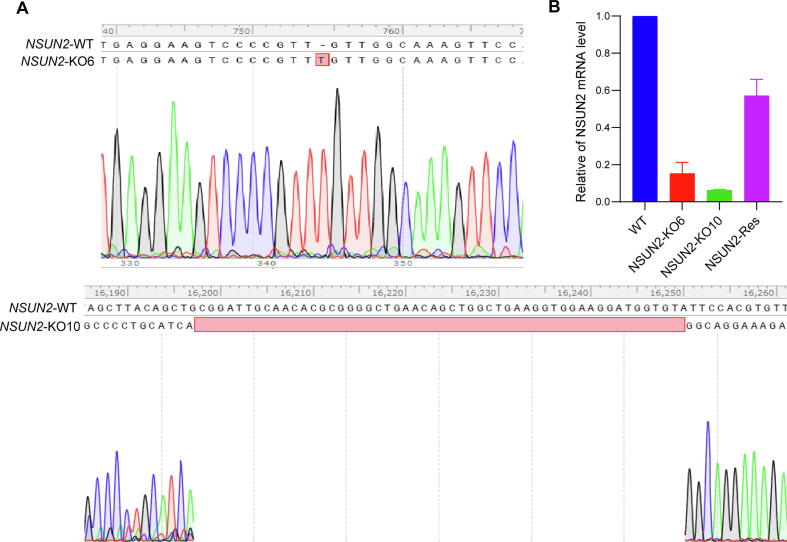
**Identification of NSUN2 knockout efficiency at the genome level and transcriptome level**. **A.** NSUN2 knockout characteristics were identified by Sanger sequencing in the QGY-7703 cell genome. **B.** The mRNA expression levels of NSUN2 in NSUN2 knockout cells (KO6/KO10) and NSUN2 reconstituted cells were evaluated by real-time PCR. WT, wilg type ; KO, knock out ; Res, rescue.

To further investigate the effect of NSUN2 on the level of active Ras, we constructed two *NSUN2*-konckout cell lines (*NSUN2*-KO6/KO10) and one *NSUN2*-rescued stable cell line (*NSUN2*-Res). The identification of *NSUN2* knockout at the genome level and the mRNA expression level are shown in Figure S3. We found that the level of active Ras protein in *NSUN2*-knockout cells was significantly decreased, which could be rescued by wild-type *NSUN2* (Figure 4D). The level of phosphorylated-Erk (p-Erk) is an important indicator of the activity of the Ras pathway. p-Erk decreased in HCC cells without changing the Erk protein level in *NSUN2*-knockout cells, and rescued by wild-type *NSUN2*. The changing of the p-Erk level cannot be rescued by mutant *NSUN2* (Figure 4E).

**Supplementary Figure S4:**
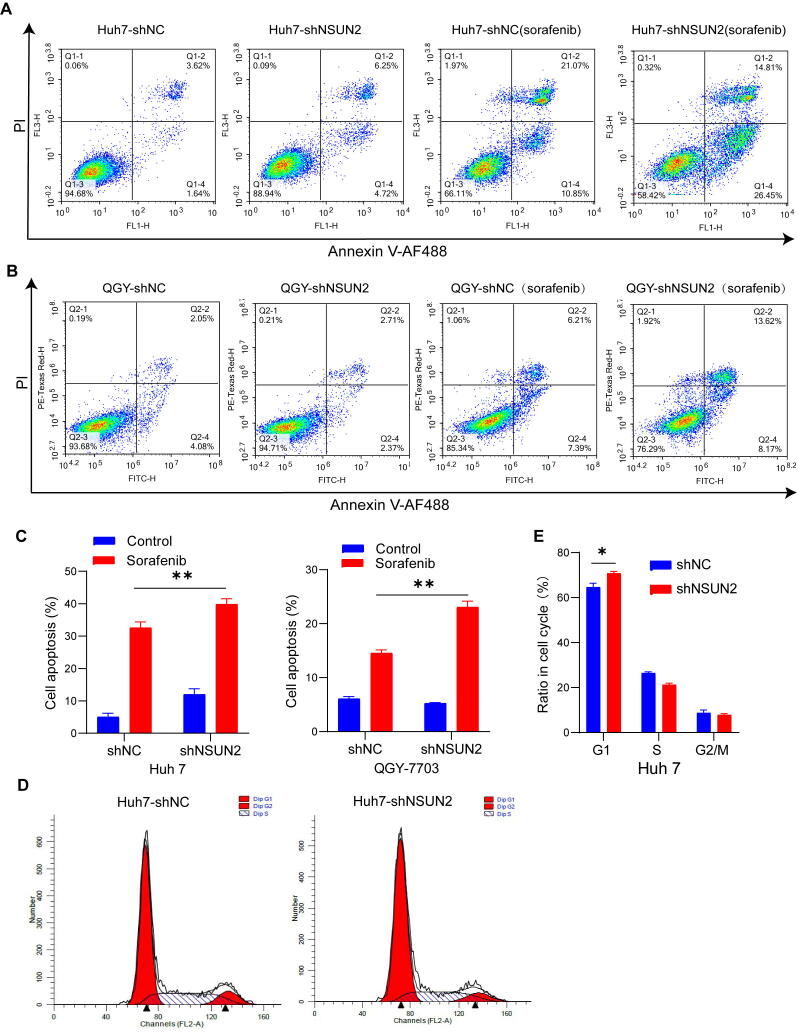
**Apoptosis and cycle analysis of NSUN2 on HCC cells**. **A.** and **B.** After sorafenib treatment, flow cytometry analyses of the apoptosis of HCC cells rate when NSUN2 knockdown. The statistical analyses of the apoptosis ratio are shown in (**C**), **, *P* < 0.01. **D.** Flow cytometry analyses of the cell cycle of Huh 7 cells. These cells were arrested in the G1 phase when NSUN2 was knocked down. The statistical analyses of the arrest ratio are shown in (**E**), *, *P* < 0.05. Statistical significance was calculated by Student’s t-test, mean ± SD. shNC, nonsense control-shRNA.

Sorafenib is a molecular inhibitor for the phosphorylation of Raf, and inhibits Ras activity, which is widely used in the systemic therapy of HCC. We investigated whether NSUN2 affects the sensitivity of sorafenib in HCC cell lines. Through flow cytometry analysis of the apoptotic HCC cells in the *NSUN2*-knockout group and the control group under sorafenib stress, the proportion of apoptotic HCC cells treated with sorafenib was significantly higher, compared to the proportion of apoptotic cells in the control group (Figure 4F). Data statistics are shown in Figure 4G. Similar results were obtained in the *NSUN2*-knockdown cells (Figure S4A and B). We observed that knockdown of *NSUN2* did not increase the apoptotic rate of QGY-7703 cells but affected that of Huh7 cells. The increased sensitivity of these cell lines to sorafenib was consistent when *NSUN2* is knocked down (Figure S4C). Additionally, we found that the down-regulation of *NSUN2* was associated with cell cycle arrest (Figure S4D and E). In summary, NSUN2 affects the sensitivity of HCC cells to sorafenib by regulating the activity of the Ras pathway.

## Discussion

In recent years, many RNA modifications have been identified [27]. As an essential epigenetic modification of RNA, m^5^C participates in different regulatory mechanisms and biological functions, especially in cancers [16], [17], [18], [19], [20], [21], [22]. In this study, the distribution characteristics of the m^5^C modification in HCC were studied. We discovered high levels of m^5^C modification and NSUN2 expression in HCC. The hypermethylated target genes (*GRB2*, *AATF*, and *RNF115*) participate in the carcinogenic pathways. NSUN2 affects the sensitivity of HCC cells to sorafenib by regulating the activity of the Ras pathway.

NSUN2 is highly expressed in multiple tumor types, such as gastric cancer and esophageal squamous cell carcinoma [18], [28], [29]. Here, we found that *NSUN2* was overexpressed in HCC. Moreover, NSUN2 in HCC tissues was strongly correlated with the high methylation and expression of target genes, including *GRB2*, *RNF115*, *AATF*, *ADAM15*, *RTN3*, and *HDGF*. Li et al. found that NSUN2 coordinates with lin-28B, a novel m^5^C recognition protein, to catalyze the m^5^C modification of *GRB2* and stabilize its mRNA expression. High levels of GRB2 promote the activation of the PI3K/Akt and Ras pathways in esophageal squamous cell carcinoma [30]. We demonstrated that NSUN2 inhibited the Ras activation and decreased the p-Erk level in HCC, which led to the increased sensitivity of HCC cells to sorafenib.

A previous study has reported that nascent RNA with m^5^C modification can regulate chromatin structures and recruit transcription factors. The m^5^C-mediated complex leads to 5-azacitidine resistance in leukemia cells, which provides new insights into the treatment of leukemia [31]. In our study, because of the critical role of NSUN2 in regulating the m^5^C modification and the expression of mRNAs related to the Ras pathway, the sensitivity of HCC cells to sorafenib was increased, which has great significance for the treatment of HCC patients. RNA epigenetics, especially m^5^C, can potentially regulate drug sensitivity.

Overall, we reveal the m^5^C landscape in HCC at a single-nucleotide resolution and verified the correlation between m^5^C-hypermethylated genes and HCC tumor characteristics. NSUN2 has been reported to be involved in various tumor-related cell processes, including affecting proliferation, apoptosis, and sorafenib sensitivity in HCC cells. Our study provides novel mechanisms for the effect of RNA epigenetic modification on HCC progression, which might help discover more effective HCC treatment targets and strategies.

## Materials and methods

### Cell lines and tissues

Human HCC cell lines (QGY-7703, Huh 7, and SMMC-7721) were cultured in Dulbecco's modified eagle medium (DMEM) supplemented with 10% fetal bovine serum and 1% penicillin/streptomycin in 5% CO_2_ at 37 °C. For sensitivity analysis of sorafenib, QGY-7703 cells were seeded in six-well plates and treated with 10 µM sorafenib for 24 h. The Huh7 cells were treated with 8 µM sorafenib for 24 h.

### Plasmids, antibodies, and real-time PCR primers

PLKO.1-shcontrol (NC), pLKO.1-shNSUN2, psPAX2, and pMD2 were used for *NSUN2* knockdown in HCC cells. The sequence of shNSUN2 was 5′-CCGGGCTGGCACAGGAGGGAATATACTCGAGTATATTCCCTCCTGTGCCAGCTTTTTG-3′. The sequence of siNSUN2 was: 5′-CACGUGUUCACUAAACCCUAUTT-3′.

The antibodies used in this study were anti-NSUN2 (Catalog No. 44056S, Cell Signaling Technology, Danvers, MA), anti-GAPDH (Catalog No. GB12002, Servicebio, Wuhan, China), anti-pErk (Catalog No. 4370s, Cell Signaling Technology), and anti-Erk (Catalog No. 4695s, Cell Signaling Technology). Real-time PCR primers for target gene quantification used in this study are as follows: *RNF115* (forward 5′-CGGCAGTCGGATAGACAATAC-3′, reverse 5′-TGTCAGGACGAGAACTTCCTC-3′), *GRB2* (forward 5′-CTGGGTGGTGAAGTTCAATTCT-3′, reverse 5′-GTTCTATGTCCCGCAGGAATATC-3′), *ADAM15* (forward 5′-CCCTGAATGTACGAGTGGCAC-3′, reverse 5′-GGAGGAAGTTTTCGAGGGTGA-3′), *AATF* (forward 5′-TCAGCCTCCCTCTTGGACA-3′, reverse 5′-TCATCAGACGATCCTGGCAGA-3′), *NSUN2* (forward 5′-GGTATCCTGAAGAACTTGCC-3′, reverse 5′-ATCTTATGATGAGGCCGCA-3′), and *GAPDH* (forward 5′-CGCTCTCTGCTCCTCCTGTTC-3′, reverse 5′-ATCCGTTGACTCCGACCTTCAC-3′).

### RNA-BisSeq and RNA-seq for HCC tissues and adjacent tissues

Tissues were frozen in liquid nitrogen and broken using Qiagen tissue lyser II (Catalog No. 69982, Qiagen, Hilden, Germany). Then, 1 ml TRIzol was added to the broken tissues, and total RNA was extracted with chloroform-isopropyl alcohol. Next, the HCC mRNAs were enriched using the Dynabeads mRNA Purification Kit (Catalog No. 61006, Ambion, Waltham, MA), and samples were treated with DNase (Catalog No. AM22222, ThermoFisher Scientific, Waltham, MA) at 37 °C for 20 min to remove genomic DNA. After DNase treatment, mRNAs were fragmented by a fragmentation reagent (Catalog No. AM8740, Ambion), and then the alcohol method was used to precipitate the samples.

After alcohol precipitation, 10 ng of mRNA samples were taken for the transcription library, and 100–200 ng of mRNA samples were taken for bisulfite treatment, according to an earlier published method [32]. Finally, we used the KAPA stranded RNA-seq library preparation kit (Catalog No. KR1139, KAPA, Potters Bar, UK) for library construction. Sequencing was performed on an Illumina HiSeq PE150 sequencing system with a paired-end 150 bp read length.

### UHPLC-MS/MS analysis

The UHPLC-MS/MS analysis was performed by a previously reported method [13]. Total RNA or mRNA (100–200 ng) was extracted from the QGY-7703 and SMMC-7721 cells, which were digested with 0.1 U nuclease P1 (Catalog No. M0660, New England Biolabs, Ipswich, MA) and 1.0 U calf intestinal alkaline phosphatase (Catalog No. 18009019, Invitrogen) at 37 °C overnight. Then, the mixture was filtered through a 3 K Omega membrane tube (Catalog No. OD010C35, PALL, New York, NY). Finally, we detected rm^5^C, rC, rU, rG, and rA using UHPLC-MS/MS.

### Immunohistochemistry

The tissues were fixed with 5 ml of formaldehyde fixative solution. Then, they were dehydrated by adding molten paraffin wax at 58 °C. Tissues were cut into 15-µm sections using a rotary microtome, suspended in a water bath at 56 °C, and mounted onto gelatin-coated histological slides. The slides were dried overnight at room temperature. Then, we performed an immunohistochemistry analysis. The samples were incubated with anti-NSUN2 (1:100) overnight at 4 °C. Finally, the expression of NSUN2 in HCC tissues was visualized under a microscope using bright-field illumination.

### Ras activation assay

RAS activity was analyzed with a Ras activation assay biochem kit (Catalog No. BK008, Cytoskeleton, Männedorf, Switzerland). The QGY-7703 cell lines containing the control group, the *NSUN2*-KO6 group, the *NSUN2*-KO10 group, and the *NSUN2*-Res group were prepared in advance, and equal concentrations of cells were collected and spread in a six-well plate. After 24 h, 500 µl of cell lysate was added to each well and centrifuged at 10,000 r/min at 4 °C for 2 min, and the supernatant protein was collected. The Bradford protein quantification kit (Catalog No. 23236, Invitrogen) was used to quantify the protein, and each group was diluted with cell lysate to equal volume and density. Then, 20 µl of whole-cell lysate was added to 5 µl of 5× sodium dodecyl sulfate (SDS) loading buffer, and the sample was boiled at 95 °C for 10 min as an input sample. The remaining samples were added with the same amount of Raf-RBD beads and rotated at 4 °C for 1 h. The beads were collected at 4 °C and centrifuged at 5000 *g* for 1 min. Then, 90% of the supernatant was removed, and the beads were cleaned three times with 500 µl of wash buffer. Finally, 1× SDS loading buffer was added, and the sample was boiled at 95 °C for 10 min as an immunoprecipitation (IP) sample. The samples were subjected to Western blot analysis, and the pan-RAS antibody was used to quantitatively identify the active Ras.

### Flow cytometry analysis

The NC group and the shNSUN2 group cells were seeded in a six-well plate. The cell confluence reached 80% through overnight culture. Then, the cells were treated with sorafenib for 24 h. The cells were harvested and washed once with precooled phosphate buffer saline (PBS). According to the protocol of the dead cell apoptosis kit (Catalog No. V13241, Invitrogen), 5× annexin-binding buffer was diluted to 1× with deionized water, and the propidium iodide (PI) staining solution was diluted to 100 µg/ml. After the buffer was prepared, the cells were resuspended in 100 µl 1× annexin-binding buffer, 5 µl Alexa Fluor 488-annexin V, and 1 µl PI (100 µg/ml). The cells were incubated for 15 min at room temperature. Then, 400 µl of 1× annexin-binding buffer was added and gently mixed before flow cytometry analysis. All the experiments were repeated at least three times.

### RNA-seq data analysis

The raw data were trimmed for adaptors by the Cutadapt software (v3.0), and low-quality bases were removed by the Trimmomatic software (v0.39) [33], [34]. The filtered clean reads were mapped to the hg19 genome with HISAT2 (v2.0) [35]. The HTSeq (v0.12.4) software was used to count reads mapped to each Ensembl gene [36]. Differentially expressed genes were calculated using DESeq2 (v1.30.1) [37]. The differential fold change cutoff was 1.2, and the false discovery rate (FDR) cutoff was 0.05.

### RNA-BisSeq data analysis

The Cutadapt and Trimmomatic software were used to trim adaptors and remove low-quality bases [33], [34]. The clean reads were mapped to the hg19 genome by meRanGh from meRanTK (v1.2.0) [38].

The m^5^C sites were called by meRanCall from meRanTK. The luciferase spike-in conversion rates were evaluated to be over 99%. The sample-credible m^5^C sites satisfied coverage depth ≥ 30, methylated cytosine depth ≥ 5, and methylation level ≥ 0.1. The differential m^5^C methylation analysis criteria comprised coverage ≥ 10 for all samples and were used to compare methylation levels between tumor and normal samples. The differential m^5^C sites were defined as follows: mean m^5^C level difference ≥ 0.05 (tumor and normal samples) and *P* < 0.05 (Wilcoxon test). The m^5^C sites were annotated using bedtools (v2.26.0) intersectBed [39].

### Pathway analysis

Hypermethylated and hypomethylated genes were used as input for DAVID (v6.8) (https://david.ncifcrf.gov/).

### Statistical analysis

Data were analyzed using the Python and GraphPad Prism (v8) software. Two-way analysis of variance and Student’s *t*-test were performed to determine statistical significance. The error bars, when present, represent the mean ± SD. The experiments were repeated at least three times independently. Statistical significance was considered at *P* < 0.05.

## Ethical statement

Human samples in this study were collected from the Biobank of the First Affiliated Hospital of Zhengzhou University, China. The study was approved by the Ethics Committee of Scientific Research and Clinical Trial of the First Affiliated Hospital of Zhengzhou University, China (Approval No. 2019-KY-0024-001). All participants provided written informed consent according to the institutional guidelines.

## Data availability

The raw sequence data reported in this study have been deposited in the Genome Sequence Archive for Human [40] at the National Genomics Data Center, Beijing Institute of Genomics, Chinese Academy of Sciences / China National Center for Bioinformation (GSA-Human: HRA001101), and are publicly accessible at https://ngdc.cncb.ac.cn/gsa-human/.

## Competing interests

The authors declare no competing interests.

## CRediT authorship contribution statement

**Dan Song:** Methodology, Validation, Investigation, Writing – original draft, Writing – review & editing. **Ke An:** Formal analysis, Data curation, Writing – original draft, Writing – review & editing. **Wenlong Zhai:** Resources, Methodology, Investigation, Writing – review & editing. **Luyao Feng:** Validation, Investigation. **Yingjie Xu:** Validation, Investigation. **Ran Sun:** Validation. **Yueqin Wang:** Methodology, Investigation. **Yun-Gui Yang:** Methodology, Supervision, Project administration. **Quancheng Kan:** Conceptualization, Project administration. **Xin Tian:** Conceptualization, Supervision, Funding acquisition, Project administration, Writing – review & editing. All authors have read and approved the final manuscript.

## Supplementary material

Supplementary Table S1 The list of mRNA m^5^C sites in HCC.
